# Weight stigma and health behaviors: evidence from the Eating in America Study

**DOI:** 10.1038/s41366-021-00814-5

**Published:** 2021-05-01

**Authors:** Kristen M. Lee, Jeffrey M. Hunger, A. Janet Tomiyama

**Affiliations:** 1 Department of Psychology, University of California, Los Angeles, CA USA; 2 Department of Psychology, Miami University, Oxford, OH USA

**Keywords:** Weight management, Obesity, Obesity, Lifestyle modification, Risk factors

## Abstract

**Background:**

Weight stigma is pervasive across the U.S. and is associated with poor health outcomes including all-cause mortality. One potential reason that weight stigma may be detrimental to health is that it begets poorer health behaviors. Therefore, the present study tested for associations between weight stigma and four health behaviors (i.e., eating behavior, alcohol use, sleep disturbance, and physical activity), while controlling for BMI and other potential confounds.

**Subjects/Methods:**

Participants (*N* = 2022) in the U.S. were recruited for the Eating in America Study using a *Qualtrics* panel between December 2019 and January 2020 and were census-matched according to national quotas for age, gender, income, race/ethnicity, and census region. Participants completed questionnaires about weight stigma, health behaviors, demographics, and anthropometric measurements. The current study employed a two-stage investigation: exploratory analyses were first performed on a random sample of the dataset (*n* = 438), then the remaining unexamined data were used to conduct confirmatory analyses that were preregistered on the Open Science Framework.

**Results:**

Controlling for BMI, weight stigma was significantly associated with greater disordered eating (*b* = 0.34, 95% CI [0.31, 0.38], *p* < 0.001), comfort eating (*b* = 0.32, 95% CI [0.25, 0.39], *p* < 0.001), sleep disturbance (*b* = 0.27, 95% CI [0.20, 0.33], *p* < 0.001), and alcohol use (*b* = 0.30, 95% CI [0.11, 0.49], *p* = 0.002), but not lower physical activity (*b* = −0.04, 95% CI [−0.13, 0.05], *p* = 0.402) for individuals across the weight spectrum. BMI and perceived weight status significantly moderated the effects of weight stigma on disordered eating and alcohol use. No gender differences were found. These confirmatory analyses partially replicated the exploratory stage 1 findings.

**Conclusions:**

This study provides preliminary evidence that weight stigma is linked to several poor health behaviors, which may impact physical health.

## Introduction

Weight stigma is pervasive. Higher weight individuals are stigmatized across many contexts, including healthcare, employment and income, education, media, and interpersonal relationships [1]. Repeated exposure to weight stigma may be consequential as considerable evidence suggests that weight stigma undermines physical health even when controlling for body mass index (BMI), which addresses the potential confound that higher adiposity begets both weight stigma and poorer health. For instance, weight discrimination predicts physiological dysregulation involving a suite of outcomes (e.g., blood pressure; inflammation; cholesterol) [2], and even mortality [3]. These findings indicate a vulnerable health profile of individuals stigmatized for their weight, particularly those with overweight or obesity. Yet, it remains unclear how weight stigma may also impact several health behaviors, which is one likely pathway through which weight stigma “gets under the skin” to generate poorer health outcomes.

Scholars have previously suggested that weight stigma may play a positive role in motivating individuals to engage in health behaviors that could lead to weight loss—presumably as a way to improve health [4]. This contention, however, is not empirically supported. Rather, stigmatizing individuals for their weight is consistently associated with negative consequences [5]. Although health behaviors are modifiable ways to improve health across the weight spectrum, individuals who experience weight stigma may cope with this mistreatment by engaging in health compromising behaviors. For instance, a person who has been stigmatized because of their size may be less likely to go to the gym, as this is an environment that puts them at risk for further stigmatization. Individuals encountering weight stigma may also eat to comfort their distress [6]. Although previous research has shown that, independent of BMI, weight stigma is associated with increased maladaptive eating behaviors and decreased motivation to exercise [7], less is known about other common health behaviors such as alcohol use and sleep. Thus, the present study examined the relationship between weight stigma and several modifiable health behaviors in a large U.S. sample. In the next section, the existing literature on weight stigma is outlined across four different health behaviors: eating behavior, physical activity, alcohol use, and sleep. Where available, findings pertaining to gender differences in these areas of research are summarized. As will become evident below, there are notable gaps in this literature that we address in the present study.

## Weight stigma and eating behavior

Eating a healthy diet helps prevent noncommunicable diseases such as diabetes, heart disease, and cancer [8]. However, weight stigma may pose one barrier to healthy eating behavior. In laboratory settings, stigmatizing individuals with higher weight (or individuals who perceived themselves as such) led to short-term increases in eating of high-fat, -sugar, and -calorie foods [9]. This eating is theorized to be a form of comfort eating due to the stress of weight stigma [6] and at least one study has found an association between weight stigma and emotional eating [10].

Weight stigma is also associated with unhealthy weight control behaviors and disordered eating such as binge eating [11]. Additionally, there is some longitudinal evidence that shows the potential long-term effects of weight stigma on adolescents’ eating and weight control practices. For example, one study found that adolescent girls who were labeled “too fat” showed increased bulimic and unhealthy weight control behaviors (e.g., using diet pills) 5 years later [12].

## Weight stigma and physical activity

Engaging in regular physical activity is associated with numerous health benefits, including lower risk for premature all-cause mortality, hypertension, Type 2 diabetes, and cardiovascular disease [13]. For higher weight individuals, weight stigma may be a barrier to engage in physical activity, though the findings are mixed. Some studies have shown that weight stigma is associated with greater exercise avoidance [14] and lower exercise self-efficacy and intentions [15]. One study found that weight bias internalization was associated with lower levels of exercise and self-efficacy, while experienced weight stigma predicted higher levels of exercise [16]. Yet other research found a positive relationship between weight discrimination and greater sedentary behavior among middle-aged and older adults [17].

## Weight stigma and alcohol use

Alcohol consumption, particularly heavy drinking, has been associated with heart disease, stroke, and some cancers [18]. There is a large body of research showing that perceived discrimination on the basis of other stigmatized identities is linked to substance use, including alcohol [19] and preliminary evidence suggests that experiencing weight stigma is associated with alcohol dependence [20]. Internalizing anti-fat attitudes may also encourage unhealthy coping mechanisms, as seen with medical students of higher weight who turn to alcohol or drugs [21]. Given the link between psychological distress and alcohol consumption [22], it is also plausible that the distress associated with weight stigma spurs unhealthy alcohol consumption.

## Weight stigma and sleep disturbance

There is a noticeable lack of research in the weight stigma literature on sleep [23], but there is reason to believe a relationship may exist given research on other stigmatized identities. For example, one systematic review found strong evidence that perceived discrimination is associated with poorer sleep, from both cross-sectional and prospective data [24]. In a large, nationally representative U.S. sample, individuals who experienced more everyday discrimination had worse objective sleep and reported greater sleep difficulties [25]. Another representative study found that perceived racial discrimination in healthcare environments independently predicted sleep disturbance [26]. Individuals with higher weight may likewise experience disturbed sleep given the chronic and pervasive nature of weight stigma.

## Weight stigma and gender differences

A significant portion of weight stigma research has focused on women with higher weight. The dearth of research on men is likely due in part to evidence that women are more likely to experience weight stigma [27]. However, recent findings indicate that weight stigma is prevalent among men as well [28]. Previous research has also shown that not all weight stigma findings related to health behaviors are uniform across genders. For example, one study found that weight teasing by family members was associated with unhealthy weight control behaviors (e.g., skipping meals, fasting) among both males and females, however only females reported extreme weight control behaviors (e.g., using laxatives, vomiting) [29]. Gender differences have also been documented in adolescence as weight-related teasing predicted greater unhealthy weight control behaviors among boys, while girls were more likely to diet [30]. In the domain of physical activity, weight stigma was associated with *more* vigorous physical activity among higher weight men, but not for women [31].

In sum, there is still much to be determined in the domain of weight stigma and health behaviors. The present study fills a critical gap in the extant literature by examining multiple health behaviors (i.e., physical activity, sleep, alcohol use, eating behavior) at once, across gender and weight status. It was hypothesized that: (1) Greater weight stigma would be associated with poorer health behaviors in the domains of physical activity, sleep disturbance, alcohol use, disordered eating, and comfort eating; (2) Weight status would moderate the association of weight stigma with health behaviors, such that the relationship would be stronger for individuals with higher BMIs and self-perceived weight. Given limited prior research, we made no directional hypotheses regarding gender differences. All a priori hypotheses and confirmatory analyses were preregistered on the Open Science Framework, https://osf.io/g4wz9?view_only=d0fafe6d7cc24846b1b1cd6b59a3783e.

## Methods

### Participants

The UCLA North General Institutional Review Board approved the present study. Participants (*N* = 2022) in the United States were recruited for the Eating in America Study through a panel on the online survey platform, *Qualtrics*, between December 2019 and January 2020. Individuals who were English speaking and at least 18 years of age were eligible to participate. Individuals were census matched according to national quotas for age, self-reported gender, income, race/ethnicity, and census region in the U.S. Eligible participants reviewed an online information sheet and gave their consent to participate.

Several steps were taken to obtain the final analytic sample in this cross-sectional study. First, a random sample (*n* = 500) was removed from the full dataset (*N* = 2022) to conduct exploratory analyses; the final exploratory sample was comprised of 438 participants after exclusion criteria were applied. Then, participants were removed from the full dataset if they met at least one of the following exclusion criteria: failed attention checks (*n* = 202), biologically implausible height (<44 inches or >90 inches; *n* = 25), weight (<55 lb or >1000 lb; *n* = 29), or BMI values (<12, or >70; *n* = 65) [32]. This yielded a sample size of 1759 after removing 263 individuals. Finally, any individuals from the exploratory sample who had not already been excluded were removed to obtain the final sample (*N* = 1327 participants). The exploratory sample was not included in the confirmatory analyses.

### Measures

Participants completed a series of self-report measures, including assessments of daily experiences with anticipated weight stigma and weight-based discrimination, physical activity, sleep disturbance, alcohol use, disordered eating, comfort eating, demographic questions, and anthropometric measurements.

#### Weight stigma

Weight stigma was assessed using a 2-item composite measure of daily anticipated and experienced weight stigma (see validation work in Online Supplementary Materials 4). Individuals indicated the frequency of anticipating weight stigma (“In your day-to-day life, how often are you concerned about or worried you will be negatively stereotyped or mistreated because of your weight?”) and experiencing weight stigma (“In your day-to-day life, how often are you treated with less respect, harassed, or discriminated against because of your weight?”) on a 4-point response scale from (Not at all–Often). A mean score was computed (α = 0.86).

#### Physical activity

Physical activity was measured with the validated [33], single item Stanford Leisure-Time Activity Categorical Item (L-CAT), which comprises six activity categories ranging from sedentary to very active based on national health recommendations determined by the American College of Sports Medicine and the American Heart Association. Each category corresponds to an intensity level based on metabolic equivalents (METs). Participants selected the category that best described their physical activity over the past month.

#### Sleep disturbance

Sleep disturbance was assessed using the 6-item Sleep Disturbance short form from the Patient-Reported Outcomes Measurement Information System item bank [34]. Participants reported sleep quality over the past week on a 5-point scale (Very Poor–Very Good) and frequency of sleep disturbance in the past week on a 5-point scale (Not at all–Very Much). A mean score was calculated for each individual, with higher scores indicating greater sleep disturbance (α = 0.90).

#### Alcohol use

Alcohol use was measured by the 3-item Alcohol Use Disorders Identification Test-C [35]. Participants indicated quantity of drinks on a typical day in the past year using a 6-point scale (0–10 or more drinks), frequency of drinking behavior in the past 30 days on a 6-point scale (Never–Six or more times a week) and frequency of binge drinking behavior in the past year on a 5-point scale (Never–Daily or Almost Daily). All three items were summed to calculate a total alcohol use score (α = 0.75).

#### Disordered eating

Disordered eating was measured using the 12-item Eating Disorder Examination Questionnaire Short (EDE-QS) [36]. Participants indicated their eating and food-related behaviors, thoughts, and feelings over the past 7 days on a 4-point rating scale (0–7 days or Not at all–Markedly). Response values range from 0 to 3, with higher scores indicating higher eating disorder symptomatology. A mean disordered eating score was calculated (α = 0.89).

#### Comfort eating

Comfort eating was defined as, “When feeling a negative emotion, some people eat more food and/or eat more unhealthy food than usual—a behavior described as comfort eating.” Participants indicated their frequency of comfort eating by selecting the number of days they engaged in comfort eating in the last 30 days. This item is distinct from disordered eating, as measured by the EDE-QS, which assesses food intake and compulsive exercise in relation to weight and body image concerns.

#### Perceived weight status

We assessed perceived weight status on a 7-point Likert scale (Very Underweight–Very Overweight) in which participants identified their perceived weight category [37].

#### Body mass index (BMI)

BMI was calculated from self-reported height and weight and categorized as: “Underweight,” <18.5, “Normal Weight,” 18.5–24.9, “Overweight,” 25–29.9, and “Obese,” >30.

#### Covariates

Covariates of age, highest level of education, gender (Woman, Man, Non-Binary/Other), race/ethnicity, and BMI were included in the analyses to test the independent effect of weight stigma on the outcomes.

### Analytic strategy

#### Exploratory analyses

We employed a two-stage investigation to run exploratory and confirmatory analyses [38]. In stage 1, exploratory analyses were conducted on a random subset (*n* = 438) to examine associations between weight stigma and the dependent variables: physical activity, sleep disturbance, alcohol use, disordered eating, and comfort eating, controlling for age, gender, race/ethnicity, education, and BMI. Linear regression models estimated for each behavioral variable found that all health behaviors except for physical activity were significantly associated with the composite weight stigma measure. These exploratory analyses appear in Online Supplementary Materials 1.

In stage 2, only the remaining unexamined data (*N* = 1327) were used to conduct the confirmatory analyses. As noted in the pre-registration, physical activity was included despite it not emerging as significant in preliminary analyses given the conflicting existing literature. In our analyses, we controlled for BMI to ensure that the relationship between weight stigma and health behaviors was not a result of confounder bias, that is, to ensure that this relationship did not emerge simply because higher BMI individuals report both greater weight stigma and poorer health behaviors. It is possible that BMI is instead a collider, or a variable that is caused by both our predictor and outcome variables [39]. Controlling for a collider can artificially create an association where one may not exist. Although greater weight stigma [40] and poorer health behaviors [41] can predict higher BMI, the results from our unadjusted models (see Table 3) are consistent with the adjusted models, suggesting that controlling for BMI does not introduce collider bias. We also examined BMI as a moderator of the relationship between weight stigma and health behaviors, as previous work has shown that effects of weight stigma exposure increase as weight increases [42].

#### Statistical analyses

Using linear regression models, the composite weight stigma score was entered as the main predictor variable and the following health behaviors were entered as the dependent variables in separate models: physical activity, sleep disturbance, alcohol use, disordered eating, and comfort eating. Age, education, race/ethnicity, gender, and BMI were included as a priori covariates. All variables were normally distributed, except for comfort eating in the exploratory and confirmatory samples. The skewed distributions were corrected using natural log transformations and all analyses were performed with the natural log values for comfort eating.

For moderation analyses, PROCESS macro (model #1) [43] was used to test the effects of BMI, perceived weight status, and gender as unique moderators on the relationship between weight stigma and all health behaviors. The same covariates were included in these models. BMI was added as a covariate in the models testing perceived weight status and gender as moderators. Given that the same pattern of results emerged for perceived weight status, these analyses appear in Online Supplementary Materials 2.

## Results

The overall sample (*N* = 1327) had a mean age of 47.7 years (*SD* = 17.2) with an average BMI of 28.0 (*SD* = 7.4; range: 12.0–68.4). Table 1 lists sample characteristics for confirmatory and exploratory samples. Those with missing data were excluded from the confirmatory analyses: BMI (*n* = 1), physical activity (*n* = 4), and comfort eating (*n* = 2). Confirmatory zero-order correlations among study variables appear in Table 2 and exploratory zero-order correlations and descriptive statistics appear in Table 4. In the confirmatory sample, 42% of participants reported weight stigma. Linear regressions estimated the associations between weight stigma and the continuous outcomes of physical activity, sleep disturbance, alcohol use, disordered eating, and comfort eating (Table 3). Adjusting for covariates of age, BMI, education, gender, and race/ethnicity, weight stigma was significantly associated with all outcomes, except for physical activity. These findings replicate those of the exploratory regression analyses from stage 1 of the investigation (see Online Supplementary Materials 1). Unadjusted models showed significant associations between weight stigma and all outcomes (Table 3). Eta-squared (*η*^2^) effect size calculations indicate that weight stigma uniquely accounted for 17.5% of the variance in disordered eating; 4.0% of the variance in sleep disturbance; 3.2% of the variance in comfort eating; and 0.7% of the variance for alcohol use.

**Table 1:** Exploratory (*N* = 438) and confirmatory (*N* = 1327) sample characteristics.

Characteristic	Exploratory		Confirmatory	
	*n*	(%)	*n*	(%)
Gender				
Women	220	50.2	669	50.4
Men	215	49.1	656	49.4
Non-binary/Other	3	0.7	2	0.2
Race/Ethnicity				
White	292	66.7	850	64.1
Black, African American	57	13.0	162	12.2
Native American, Eskimo, Aleut	4	0.9	19	1.4
Hispanic, Latino/a	52	11.9	201	15.1
Asian, Asian American	24	5.5	72	5.4
Native Hawaiian or Pacific Islander	1	0.2	1	0.1
Biracial/Multiracial	7	1.6	17	1.3
Other	1	0.2	5	0.4
Education				
Less than high school	11	2.5	27	2.0
High school diploma or GED	88	20.1	264	19.9
Some college, but no degree	106	24.2	336	25.3
Associate degree	60	13.7	178	13.4
Bachelor’s degree	113	25.8	316	23.8
Master’s degree	48	11.0	153	11.5
Doctorate or professional degree	12	2.7	53	4.0
Income				
Less than $25,000	77	17.6	223	16.8
$25,000–$49,999	102	23.3	287	21.6
$50,000–$74,999	84	19.2	263	19.8
$75,000–$99,000	60	13.7	198	14.9
$100,000–$149,999	64	14.6	207	15.6
$150,000–$199,999	31	7.1	71	5.4
Over $200,000	20	4.6	78	5.9
BMI category				
“Underweight” (<18.5)	11	2.5	49	3.7
“Normal weight” (18.5–24.9)	142	32.4	455	34.3
“Overweight” (25–29.9)	147	33.6	406	30.6
“Obesity” (>30)	138	31.5	417	31.4
Perceived weight status				
Very underweight	3	0.7	10	0.8
Underweight	19	4.3	48	3.6
Slightly underweight	28	6.4	69	5.2
About the right weight	131	29.9	426	32.1
Slightly overweight	155	35.4	439	33.1
Overweight	79	18.0	264	19.9
Very overweight	23	5.3	71	5.4

**Table 2:** Zero-order confirmatory correlations among key variables.

Measure	1	2	3	4	5
1. Weight stigma *M* = 1.76 (0.86)					
2. Physical activity *M* = 2.58 (1.40)	−0.085**				
3. Sleep disturbance *M* = 2.65 (1.03)	0.276***	−0.121***			
4. Alcohol use *M* = 2.79 (2.81)	0.103***	0.040	0.120***		
5. Disordered eating *M* = 0.77 (0.63)	0.544***	0.004	0.328***	0.220***	
6. Comfort eating *M* = 6.64 (8.18)	0.319***	−0.118***	0.253***	0.170***	0.414***

Missing data excluded for physical activity (*n* = 4); comfort eating (*n* = 2). Natural log values for comfort eating were used in the analyses.

*p < 0.05; **p < 0.01; ***p < 0.001.

**Table 3:** Regression coefficients of weight stigma on health behaviors unadjusted and adjusted for age, gender, education, race/ethnicity, and BMI .

	Adjusted coefficient					Unadjusted coefficient				
	*b*	*SE*	*β*	95% CI of *b*	*p*	*b*	*SE*	*β*	95% CI of *b*	*p*
Sleep disturbance										
Weight stigma	0.27	0.03	0.22	0.20, 0.33	**<0.001**	0.33	0.03	0.28	0.27, 0.39	**<0.001**
Age	−0.01	0.002	−0.16	−0.01, −0.01	**<0.001**					
Gender	−0.13	0.05	−0.07	−0.24, −0.03	**0.015**					
Race/Ethnicity	−0.001	0.02	−0.001	−0.04, 0.04	0.979					
Education	−0.05	0.02	−0.08	−0.09, −0.02	**0.002**					
BMI	0.001	0.004	0.01	−0.01, 0.01	0.764					
Alcohol use										
Weight stigma	0.30	0.10	0.09	0.11, 0.49	**0.002**	0.34	0.09	0.10	0.16, 0.51	**<0.001**
Age	−0.03	0.01	−0.17	−0.04, −0.02	**<0.001**					
Gender	0.90	0.15	0.16	0.60, 1.20	**<0.001**					
Race/Ethnicity	0.02	0.06	0.01	−0.10, 0.13	0.771					
Education	0.10	0.05	0.05	0.00, 0.20	**0.049**					
BMI	−0.03	0.01	−0.08	−0.05, −0.01	**0.007**					
Disordered eating										
Weight stigma	0.34	0.02	0.47	0.31, 0.38	**<0.001**	0.40	0.02	0.54	0.37, 0.43	**<0.001**
Age	−0.01	0.001	−0.17	−0.01, −0.004	**<0.001**					
Gender	−0.14	0.03	−0.11	−0.20, −0.08	**<0.001**					
Race/Ethnicity	−0.01	0.01	−0.02	−0.03, 0.01	0.473					
Education	0.03	0.01	0.07	0.01, 0.05	**0.003**					
BMI	0.01	0.002	0.09	0.004, 0.01	**<0.001**					
Comfort eating										
Weight stigma	0.32	0.04	0.25	0.25, 0.39	**<0.001**	0.40	0.03	0.32	0.34, 0.47	**<0.001**
Age	−0.01	0.002	−0.15	−0.01, −0.005	**<0.001**					
Gender	0.02	0.06	0.01	−0.10, 0.13	0.787					
Race/Ethnicity	−0.003	0.02	−0.004	−0.05, 0.04	0.882					
Education	0.05	0.02	0.07	0.01, 0.09	**0.009**					
BMI	0.01	0.004	0.10	0.01, 0.02	**0.001**					
Physical activity										
Weight stigma	−0.04	0.05	−0.02	−0.13, 0.05	0.402	−0.14	0.04	−0.09	−0.23, −0.05	**0.002**
Age	−0.01	0.003	−0.08	−0.01, −0.002	**0.006**					
Gender	0.18	0.08	0.07	0.03, 0.33	**0.016**					
Race/Ethnicity	−0.11	0.03	−0.11	−0.16, −0.05	**<0.001**					
Education	0.10	0.02	0.10	0.05, 0.14	**<0.001**					
BMI	−0.04	0.01	−0.20	−0.05, −0.03	**<0.001**					

Bold indicates statistical significance (*p* < 0.05). *SE* = standard error; *b* = unstandardized regression coefficient; *β* = standardized regression coefficient. Natural log values for comfort eating were used in the analyses. Gender was coded as: 1 = Woman, 2 = Man, 3 = Non-binary/Other. Race/ethnicity was coded as: 1 = White, 2 = Black/African American, 3 = Native American/Eskimo/Aleut, 4 = Hispanic/Latinx, 5 = Asian/Asian-American, 6 = Native Hawaiian or Pacific Islander, 7 = Biracial/Multiracial, 8 = Other. Education was coded as: 1 = Less than high school, 2 = High school diploma or equivalent, 3 = Some college, but no degree, 4 = Associate degree, 5 = Bachelor’s degree, 6 = Master’s degree, 7 = Doctorate or professional degree such as JD/MD. Age and BMI were entered as continuous variables.

**Fig. 1:**
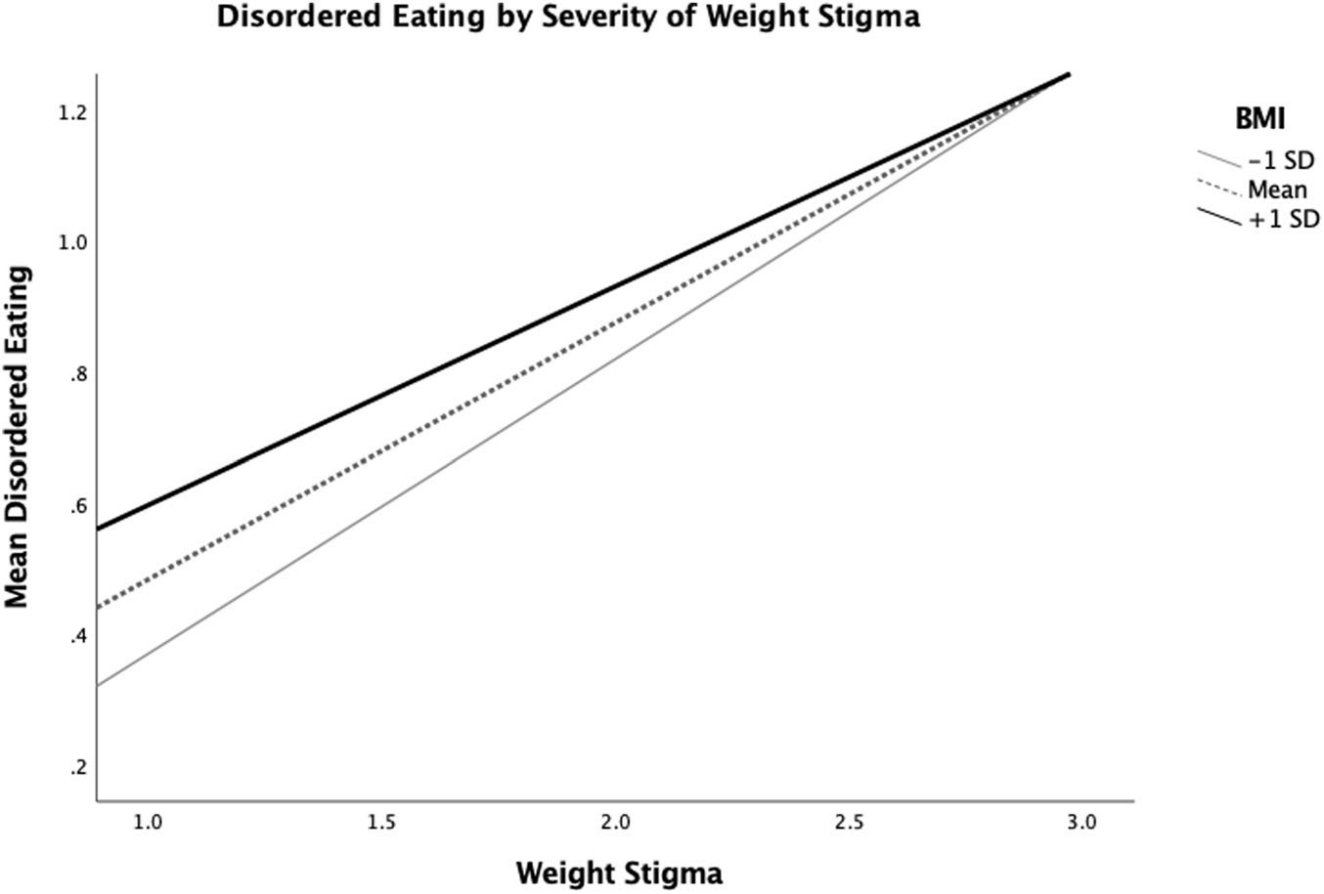
Mean disordered eating by weight stigma, according to BMI. Mean BMI was 28.0 (*SD* = 7.4).

**Fig. 2:**
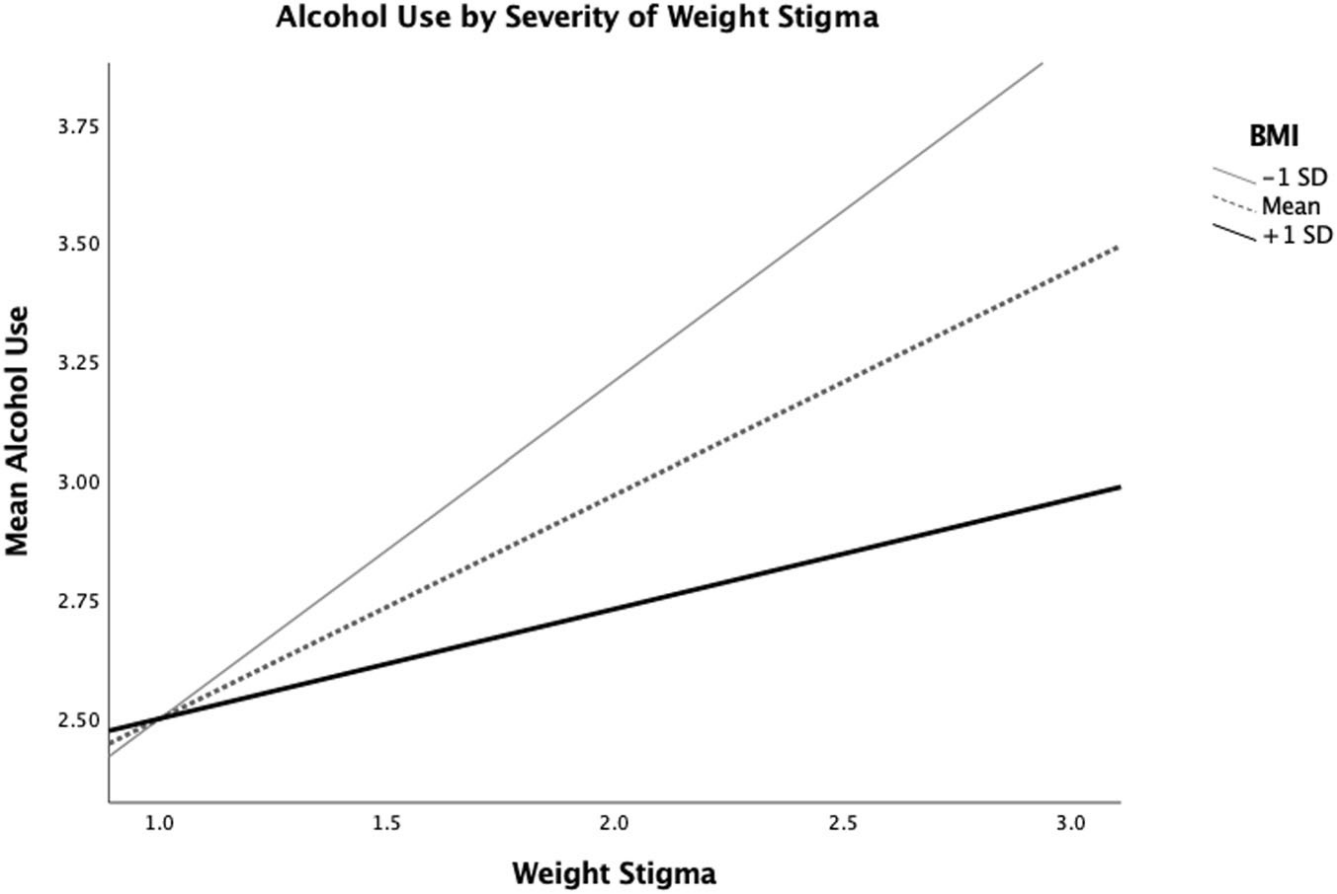
Mean alcohol use by weight stigma, according to BMI. Mean BMI was 28.0 (*SD* = 7.4).

Moderation models tested whether BMI modified the association of weight stigma with outcomes. BMI significantly moderated the association of weight stigma with disordered eating (*b* = −0.01, *SE* = 0.002, 95% CI [−0.01, −0.005], *t*(1319) = −4.45, *p* < 0.001; Fig. 1) and alcohol use (*b* = −0.03, *SE* = 0.01, 95% CI [−0.05, −0.01], *t*(1319) = −2.72, *p* = 0.007; Fig. 2), but not the other health behaviors (see Online Supplementary Materials 3, Table 3). Using the Johnson–Neyman technique, the relationship between weight stigma and disordered eating appeared to be weaker as BMI increased, up to a BMI of 55.5 (“severe obesity”) and between weight stigma and alcohol, up to a BMI of 32.09 (“obesity”).

Lastly, exploratory moderation analyses were conducted to test the effect of gender on the relationship between weight stigma and health behaviors. No significant interaction effects were observed (see Online Supplementary Materials 3, Table 4).

**Table 4:** Zero-order exploratory correlations among key variables.

Measure	1	2	3	4	5
1. Weight stigma *M* = 1.78 (0.86)					
2. Physical activity *M* = 2.69 (1.41)	−0.085**				
3. Sleep disturbance *M* = 2.65 (1.01)	0.276***	−0.141**			
4. Alcohol use *M* = 2.76 (2.71)	0.153**	0.097*	0.189***		
5. Disordered eating *M* = 0.72 (0.60)	0.475***	−0.066	0.285***	0.250***	
6. Comfort eating *M* = 5.97 (7.90)	0.350***	−0.116*	0.162**	0.125**	0.368***

Missing data excluded for physical activity (*n* = 3). Natural log values for comfort eating were used for the analyses.

*p < 0.05; **p < 0.01; ***p < 0.001.

## Discussion

The present study employed a two-stage research investigation to examine the relationship between weight stigma and several health behaviors in a large sample of U.S. adults. As predicted, weight stigma was significantly associated with greater disordered eating, comfort eating, alcohol use, and sleep disturbance, after controlling for covariates. No such relationship was observed for physical activity.

Targeting health behaviors (e.g., eating) to achieve weight loss is common in weight-focused health promotion policies. These policies assume that individuals can improve their health by losing weight, employing weight stigma as one strategy for motivating behavior change [5]. However, our findings indicate that weight stigma is associated with poorer health behaviors, independent of BMI. Given that physical health and weight are largely shaped by factors outside of an individual’s control (i.e., genetics and social determinants like socioeconomic status) [5], it is concerning that multiple behaviors, for which individuals have some control over, may be undermined by weight stigma.

Furthermore, a lower BMI may not necessarily be protective against weight stigma. In our sample, individuals across the weight spectrum, not only those with overweight or obese BMIs, reported weight stigma. In fact, moderation analyses indicated that individuals with *lower* BMIs showed greater disordered eating and alcohol use in the face of weight stigma. These results emerged despite individuals with higher weight reporting greater daily weight stigma. One explanation for the observed differences in health behavioral outcomes across the weight spectrum is that infrequent health behaviors may be less likely to be enacted as coping strategies. For instance, previous research has shown that alcohol use decreases as BMI increases among females with higher weight [44]. Thus, using food, instead of alcohol, may be the more common coping strategy among individuals with higher BMIs, as previous research suggests [45]. Nonetheless, the sizes of the moderation effects were very small, with some confidence intervals functionally at zero, and thus further interpretation of the present findings should only be done with caution.

Prior research has found conflicting evidence for the relationship between weight stigma and physical activity. Some studies have found that greater weight stigma is associated with short-term increases in reported exercise behavior [16, 46]. Others have shown that weight stigma is positively correlated with increased exercise avoidance, but has no direct link to self-reported exercise [14, 47]. The current study adds to the latter base of evidence showing no relationship between weight stigma and physical activity. One possible explanation is that participants were asked about their daily experiences with weight stigma, which may not correspond to their level of physical activity over the past month. Ecological momentary assessment methodology may provide better insight into this relationship, as demonstrated by Vartanian et al. who examined health motivations following stigmatizing events in daily life [48].

Despite emerging evidence that weight stigma is prevalent among men [28], there is a lack of research on men’s health outcomes related to weight stigma. In this study, moderation by gender was not observed for any outcome. These results are consistent with previous research reporting no gender differences in poor health outcomes such as mortality and obesity due to weight stigma [3, 49]. Men may also feel pressured to meet societal body standards and thus may display the same magnitude of associations between weight stigma and health behaviors. It is recommended that the null gender findings are interpreted with caution, as more research is needed.

How might weight stigma influence an individual’s health behaviors? One potential mechanism is stress. Previous work suggests that weight stigma is stressful [6, 50] and experimental lab studies manipulating weight stigma have shown that individuals with higher weight, as well as those who perceive themselves as overweight, show elevated levels of the stress hormone cortisol following exposure to a weight-stigmatizing event [51, 52]. Additionally, some research has found that individuals who experience more weight-based discrimination have higher hair cortisol levels—a finding most pronounced in those at the highest BMI [53]. Individuals who experience greater stress may engage in more unhealthy coping behaviors. Indeed, stress can drive changes in behaviors such as eating, physical activity, and sleep [54]. For example, Tataranni et al. administered synthetic cortisol vs. placebo and found greater food consumption in the cortisol group [55]. This early work is supported by accumulating evidence that cortisol is associated with increased caloric intake and greater abdominal fat storage [56]. The health behavior pathway may not be independent from that of stress but rather reflect a serial mediation model, wherein weight stigma increases stress that in turn causes decrements in health behaviors.

The present study contributes to the weight stigma literature in several ways. As noted, a key strength of this study is the assessment of several health behaviors within a large, national census-matched sample. Previous studies that have examined weight stigma in relation to different health behaviors have often had small sample sizes or were limited to female subjects. Thus, the present study may provide more generalizable information about health behaviors in the U.S. A related strength of the study is that higher BMI scores were well-represented in the sample, with 31.4% meeting BMI criteria for obesity. This is a closer estimate of the proportion of the American population that is classified with obesity (42%) compared to previous studies [57]. Therefore, there is greater confidence that these findings reflect the experiences of individuals with obesity in the population, aiding generalizability. Lastly, we enhance reproducibility by presenting a two-stage research program of exploratory and confirmatory analyses based on recommended open science practices.

There are some limitations to consider. First, a composite weight stigma score was used due to survey constraints. While early tests indicate good construct validity, additional psychometric testing is warranted (see Online Supplementary Materials 4). Another limitation is the use of self-reported weight, which is subject to inaccuracies. Additionally, the data collection period overlapped with winter holidays. Individuals may have made new year’s health resolutions, and therefore the self-reported health behaviors may be more indicative of newly established goals rather than typical health habits. However, such resolutions would likely dampen, rather than magnify, the relationship between weight stigma and poor health behaviors. Lastly, the study is cross-sectional and therefore causal direction cannot be determined. Weight stigma may operate as a feedback loop that leads to weight gain through certain behaviors such as comfort eating [6], but further investigation is required. Given survey constraints, weight bias internalization was not assessed. Future research should build on these findings to determine the potential role of weight bias internalization in these health behaviors.

Despite these limitations, these study’s findings show that weight stigma is significantly associated with several health behaviors. If future research confirms that this is indeed a causal relationship, weight stigma could cumulatively undermine physical health over time. Taken together, these findings highlight weight stigma as a potential barrier to healthy behaviors, and suggest that one strategy to improve population health may be to reduce weight stigma. Though more research is needed, it may be important to employ more weight-inclusive approaches to health promotion, such as removing stigmatizing language or weight outcomes from health policies and program objectives [5].

## Supplementary information

Electronic Supplementary Materials
