# Supplementary material for: Weight stigma and health behaviors: evidence from the Eating in America Study
Source: Int J Obes (Lond). 2021 May 1;45(7):1499–509. doi: 10.1038/s41366-021-00814-5 (PMC8236399; doi:10.1038/s41366-021-00814-5)
Supplement: Supplementary file 1 — Electronic Supplementary Materials [file 41366_2021_814_MOESM1_ESM.docx]

**Online Supplementary Materials 1**

| **Table 1** | |  |  |  |  |  |  |  |  |  |  |  | |
| --- | --- | --- | --- | --- | --- | --- | --- | --- | --- | --- | --- | --- | --- |
| *Exploratory Analyses: Regression Coefficients of Weight Stigma on Health Behaviors Unadjusted and Adjusted for Age, Gender, Education, Race/Ethnicity, and BMI (n = 438)* | | | | | | | | | | | | | |
|  | |  |  |  |  |  |  |  |  |  |  |  | |
|  | | *Adjusted coefficient* | | | | | *Unadjusted coefficient* | | | | | |  |
|  | | *b* | *SE* | *β* | 95% CI of *b* | *p* | *b* | *SE* | *β* | 95% CI of *b* | *p* |  | |
| Sleep disturbance | | | | | | | | | | | | |  |
| Weight Stigma | 0.22 | 0.06 | 0.19 | [0.10, 0.34] | **<.001** | 0.27 | 0.06 | 0.23 | [0.16, 0.38] | **<.001** |  | |  |
| Age | | -0.01 | 0.003 | -0.14 | [-0.01, -0.002] | **.008** |  |  |  |  |  |  | |
| Gender | | -0.22 | 0.09 | -0.11 | [-0.39, -0.04] | **.019** |  |  |  |  |  |  | |
| Race/Ethnicity | | -0.01 | 0.04 | -0.01 | [-0.08, 0.06] | .787 |  |  |  |  |  |  | |
| Education | | -0.10 | 0.03 | -0.15 | [-0.15, -0.04] | **.001** |  |  |  |  |  |  | |
| BMI | | 0.004 | 0.01 | 0.02 | [-0.01, 0.02] | .629 |  |  |  |  |  |  | |
| Alcohol use | | | | | | | | | | | | |  |
| Weight Stigma | | 0.41 | 0.16 | 0.13 | [0.09, 0.73] | **.011** | 0.49 | 0.15 | 0.16 | [0.19, 0.78] | **.001** |  | |
| Age | | -0.02 | 0.01 | -0.12 | [-0.04, -0.002] | **.030** |  |  |  |  |  |  | |
| Gender | | 1.00 | 0.25 | 0.19 | [0.51, 1.49] | **<.001** |  |  |  |  |  |  | |
| Race/Ethnicity | | -0.08 | 0.10 | -0.05 | [-0.27, 0.10] | .380 |  |  |  |  |  |  | |
| Education | | 0.03 | 0.08 | 0.02 | [-0.12, 0.19] | .690 |  |  |  |  |  |  | |
| BMI | | 0.00 | 0.02 | 0.001 | [-0.04, 0.04] | .985 |  |  |  |  |  |  | |
| Disordered eating | | | | | | | | | | | | |  |
| Weight Stigma | | 0.24 | 0.03 | 0.35 | [0.18, 0.31] | **<.001** | 0.33 | 0.03 | 0.48 | [0.27, 0.39] | **<.001** |  | |
| Age | | -0.01 | 0.002 | -0.23 | [-0.01, -0.01] | **<.001** |  |  |  |  |  |  | |
| Gender | | -0.11 | 0.05 | -0.09 | [-0.20, -0.02] | **.022** |  |  |  |  |  |  | |
| Race/Ethnicity | | -0.04 | 0.02 | -0.10 | [-0.08, -0.003] | **.033** |  |  |  |  |  |  | |
| Education | | 0.02 | 0.02 | 0.05 | [-0.01, 0.05] | .197 |  |  |  |  |  |  | |
| BMI | | 0.02 | 0.004 | 0.22 | [0.01, 0.03] | **<.001** |  |  |  |  |  |  | |
| Comfort eating | | | | | | | | | | | | |  |
| Weight Stigma | | 0.41 | 0.06 | 0.32 | [0.29, 0.54] | **<.001** | 0.46 | 0.06 | 0.35 | [0.34, 0.57] | **<.001** |  | |
| Age | | -0.003 | 0.003 | -0.05 | [-0.01, 0.004] | .364 |  |  |  |  |  |  | |
| Gender | | -0.09 | 0.10 | -0.04 | [-0.28, 0.11] | .371 |  |  |  |  |  |  | |
| Race/Ethnicity | | -0.05 | 0.04 | -0.07 | [-0.13, 0.02] | .160 |  |  |  |  |  |  | |
| Education | | -0.02 | 0.03 | -0.02 | [-0.08, 0.04] | .585 |  |  |  |  |  |  | |
| BMI | | 0.01 | 0.01 | 0.08 | [-0.002, 0.03] | .085 |  |  |  |  |  |  | |
| Physical activity | | | | | | | | | | | | |  |
| Weight Stigma | | -0.09 | 0.09 | -0.05 | [-0.26, 0.08] | .303 | -0.14 | 0.08 | -0.08 | [-0.29, 0.02] | .079 |  | |
| Age | | -0.002 | 0.004 | -0.03 | [-0.01, 0.01] | .625 |  |  |  |  |  |  | |
| Gender | | 0.27 | 0.13 | 0.10 | [0.01, 0.53] | **.043** |  |  |  |  |  |  | |
| Race/Ethnicity | | -0.04 | 0.05 | -0.04 | [-0.14, 0.06] | .446 |  |  |  |  |  |  | |
| Education | | 0.11 | 0.04 | 0.12 | [0.02, 0.19] | **.012** |  |  |  |  |  |  | |
| BMI | | -0.02 | 0.01 | -0.10 | [-0.04, -0.001] | **.042** |  |  |  |  |  |  | |
| *Note.* Bold indicates statistical significance (*p* < .05). *SE* = standard error; *b* = unstandardized regression coefficient; *β =* standardized regression coefficient. Gender was coded as 1 = Woman, 2 = Man, 3 = Non-binary/Other. Race/ethnicity was coded as 1 = White, 2 = Black/African American, 3 = Native American/Eskimo/Aleut, 4 = Hispanic/Latinx, 5 = Asian/Asian-American, 6 = Native Hawaiian or Pacific Islander, 7 = Biracial/Multiracial, 8 = Other. Education was coded as 1 = Less than high school, 2 = High school diploma or equivalent, 3 = Some college, but no degree, 4 = Associate degree, 5 = Bachelor’s degrees, 6 = Master’s degree, 7 = Doctorate or professional degree such as JD/MD. Age and BMI were entered as continuous variables. Natural log values for comfort eating were used in the analyses. | | | | | | | | | | | | | |

**Online Supplementary Materials 2**

| **Table 2** |  |  |  |  |  |  |
| --- | --- | --- | --- | --- | --- | --- |
| *Perceived Weight Status x Weight Stigma Interaction Effect* | | |  |  |  |  |
|  |  |  |  |  |  |  |
|  | | *b* | *SE* | *t* | 95% CI of *b* | *p* |
| Sleep disturbance |  |  |  |  |  |  |
|  | Weight Stigma | 0.27 | 0.03 | 7.73 | [0.20, 0.34] | **<.001** |
|  | Perceived Weight Status | -0.01 | 0.03 | -0.17 | [-0.07, 0.06] | .862 |
|  | PWS x Weight Stigma | -0.03 | 0.03 | -1.00 | [-0.07, 0.02] | .317 |
|  | Age | -0.01 | 0.00 | -5.22 | [-0.013, -0.006] | **<.001** |
|  | Gender | -0.13 | 0.05 | -2.47 | [-0.24, -0.03] | **.014** |
|  | Race/Ethnicity | 0.00 | 0.02 | -0.01 | [-0.04, 0.04] | .992 |
|  | Education | -0.05 | 0.02 | -2.93 | [-0.09, -0.02] | **.003** |
|  | BMI | 0.00 | 0.01 | 0.59 | [-0.01, 0.01] | .555 |
| Alcohol use |  |  |  |  |  |  |
|  | Weight Stigma | 0.32 | 0.10 | 3.27 | [0.13, 0.51] | **.001** |
|  | Perceived Weight Status | 0.04 | 0.09 | 0.41 | [-0.14, 0.21] | .682 |
|  | PWS x Weight Stigma | -0.18 | 0.07 | -2.59 | [-0.32, -0.04] | **.010** |
|  | Age | -0.03 | 0.01 | -5.53 | [-0.04, -0.02] | **<.001** |
|  | Gender | 0.89 | 0.15 | 5.85 | [0.59, 1.19] | **<.001** |
|  | Race/Ethnicity | 0.02 | 0.06 | 0.33 | [-0.09, 0.13] | .742 |
|  | Education | 0.10 | 0.05 | 2.03 | [0.003, 0.20] | **.043** |
|  | BMI | -0.02 | 0.01 | -1.71 | [-0.05, 0.004] | .088 |
| Disordered eating |  |  |  |  |  |  |
|  | Weight Stigma | 0.34 | 0.02 | 18.41 | [0.30, 0.37] | **<.001** |
|  | Perceived Weight Status | 0.12 | 0.02 | 7.18 | [0.09, 0.15] | **<.001** |
|  | PWS x Weight Stigma | -0.04 | 0.01 | -3.32 | [-0.07, -0.02] | **.001** |
|  | Age | -0.01 | 0.00 | -7.05 | [-0.01, -0.005] | **<.001** |
|  | Gender | -0.12 | 0.03 | -4.20 | [-0.18, -0.06] | **<.001** |
|  | Race/Ethnicity | -0.01 | 0.01 | -0.71 | [-0.03, 0.01] | .481 |
|  | Education | 0.02 | 0.01 | 2.04 | [0.001, 0.04] | **.041** |
|  | BMI | 0.00 | 0.00 | -0.66 | [-0.01, 0.003] | .512 |
| Comfort eating |  |  |  |  |  |  |
|  | Weight Stigma | 0.31 | 0.04 | 8.41 | [0.23, 0.38] | **<.001** |
|  | Perceived Weight Status | 0.13 | 0.03 | 3.96 | [0.07, 0.20] | **<.001** |
|  | PWS x Weight Stigma | -0.01 | 0.03 | -0.49 | [-0.06, 0.04] | .624 |
|  | Age | -0.01 | 0.002 | -5.24 | [-0.014, -0.01] | **<.001** |
|  | Gender | 0.04 | 0.06 | 0.70 | [-0.07, 0.15] | .483 |
|  | Race/Ethnicity | -0.003 | 0.02 | -0.15 | [-0.05, 0.04] | .879 |
|  | Education | 0.04 | 0.02 | 2.02 | [0.001, 0.08] | **.043** |
|  | BMI | 0.002 | 0.01 | 0.34 | [-0.009, 0.01] | .732 |
| Physical activity |  |  |  |  |  |  |
|  | Weight Stigma | -0.03 | 0.05 | -0.72 | [-0.13, 0.06] | .472 |
|  | Perceived Weight Status | -0.07 | 0.04 | -1.58 | [-0.16, 0.02] | .115 |
|  | PWS x Weight Stigma | 0.02 | 0.03 | 0.61 | [-0.05, 0.09] | .545 |
|  | Age | -0.01 | 0.00 | -2.58 | [-0.01, -0.002] | **.010** |
|  | Gender | 0.17 | 0.08 | 2.26 | [0.02, 0.32] | **.024** |
|  | Race/Ethnicity | -0.11 | 0.03 | -3.70 | [-0.16, -0.05] | **<.001** |
|  | Education | 0.10 | 0.03 | 3.97 | [0.05, 0.15] | **<.001** |
|  | BMI | -0.03 | 0.01 | -4.46 | [-0.05, -0.02] | **<.001** |
|  |  |  |  |  |  |  |
| *Note.* Interaction effects controlling for age, gender, race/ethnicity, education, BMI. Gender was coded as 1 = Woman, 2 = Man, 3 = Non-binary/Other. Race/ethnicity was coded as 1 = White, 2 = Black/African American, 3 = Native American/Eskimo/Aleut, 4 = Hispanic/Latinx, 5 = Asian/Asian-American, 6 = Native Hawaiian or Pacific Islander, 7 = Biracial/Multiracial, 8 = Other. Education was coded as 1 = Less than high school, 2 = High school diploma or equivalent, 3 = Some college, but no degree, 4 = Associate degree, 5 = Bachelor’s degree, 6 = Master’s degree, 7 = Doctorate or professional degree such as JD/MD. Bold indicates statistical significance (*p* < .05). PWS = Perceived Weight Status; *SE* = standard error; *b* = unstandardized regression coefficient. Natural log values for comfort eating were used in the analyses. | | | | | | |

**Online Supplementary Materials 3**

| **Table 3** |  |  |  |  |  |  |  |
| --- | --- | --- | --- | --- | --- | --- | --- |
| *BMI x Weight Stigma Interaction Effect* | |  |  |  |  |  |  |
|  | *b* | *SE* | *t* | 95% CI of *b* | *p* |  |  |
| Sleep disturbance |  |  |  |  |  |  |  |
| Weight Stigma | 0.27 | 0.03 | 7.77 | [0.20, 0.34] | **<.001** |  |  |
| BMI | 0.005 | 0.004 | 1.02 | [-0.004, 0.01] | .310 |  |  |
| BMI x Weight Stigma | -0.01 | 0.004 | -1.57 | [-0.01, 0.001] | .117 |  |  |
| Age | -0.01 | 0.002 | -5.31 | [-0.013, -0.006] | **<.001** |  |  |
| Gender | -0.14 | 0.05 | -2.54 | [-0.24, -0.03] | **.011** |  |  |
| Race/Ethnicity | -0.001 | 0.02 | -0.04 | [-0.04, 0.04] | .965 |  |  |
| Education | -0.05 | 0.02 | -3.02 | [-0.09, -0.02] | **.003** |  |  |
| Alcohol use |  |  |  |  |  |  |  |
| Weight Stigma | 0.31 | 0.10 | 3.27 | [0.13, 0.50] | **.001** |  |  |
| BMI | -0.01 | 0.01 | -1.08 | [-0.04, 0.01] | .280 |  |  |
| BMI x Weight Stigma | -0.03 | 0.01 | -2.72 | [-0.05, -0.01] | **.007** |  |  |
| Age | -0.03 | 0.01 | -5.62 | [-0.04, -0.02] | **<.001** |  |  |
| Gender | 0.87 | 0.15 | 5.72 | [0.57, 1.17] | **<.001** |  |  |
| Race/Ethnicity | 0.01 | 0.06 | 0.26 | [-0.10, 0.13] | .795 |  |  |
| Education | 0.10 | 0.05 | 2.02 | [0.003, 0.20] | **.043** |  |  |
| Disordered eating |  |  |  |  |  |  |  |
| Weight Stigma | 0.35 | 0.02 | 18.97 | [0.31, 0.38] | **<.001** |  |  |
| BMI | 0.01 | 0.002 | 5.43 | [0.01, 0.02] | **<.001** |  |  |
| BMI x Weight Stigma | -0.01 | 0.002 | -4.45 | [-0.01, -0.005 | **<.001** |  |  |
| Age | -0.01 | 0.001 | -6.37 | [-0.01, -0.004] | **<.001** |  |  |
| Gender | -0.15 | 0.03 | -5.19 | [-0.21, -0.09] | **<.001** |  |  |
| Race/Ethnicity | -0.01 | 0.01 | -0.77 | [-0.03, 0.01] | .439 |  |  |
| Education | 0.03 | 0.01 | 3.05 | [0.01, 0.05] | **.002** |  |  |
| Comfort eating |  |  |  |  |  |  |  |
| Weight Stigma | 0.32 | 0.04 | 8.88 | [0.25, 0.39] | **<.001** |  |  |
| BMI | 0.02 | 0.005 | 3.88 | [0.01, 0.03] | **<.001** |  |  |
| BMI x Weight Stigma | -0.01 | 0.004 | -1.82 | [-0.01, 0.001] | .069 |  |  |
| Age | -0.01 | 0.002 | -4.84 | [-0.01, -0.006] | **<.001** |  |  |
| Gender | 0.01 | 0.06 | 0.12 | [-0.11, 0.12] | .902 |  |  |
| Race/Ethnicity | -0.004 | 0.02 | -0.17 | [-0.05, 0.04] | .866 |  |  |
| Education | 0.05 | 0.02 | 2.67 | [0.01, 0.09] | **.008** |  |  |
| Physical activity |  |  |  |  |  |  |  |
| Weight Stigma | -0.17 | 0.15 | -1.09 | [-0.47, 0.13] | .278 |  |  |
| BMI | -0.05 | 0.01 | -3.62 | [-0.07, -0.02] | **<.001** |  |  |
| BMI x Weight Stigma | 0.004 | 0.01 | 0.87 | [-0.01, 0.01] | .386 |  |  |
| Age | -0.01 | 0.003 | -2.74 | [-0.01, -0.002] | **.006** |  |  |
| Gender | 0.19 | 0.08 | 2.48 | [0.04, 0.34] | **.013** |  |  |
| Race/Ethnicity | -0.11 | 0.03 | -3.68 | [-0.16, -0.05] | **<.001** |  |  |
| Education | 0.09 | 0.02 | 3.79 | [0.05, 0.14] | **<.001** |  |  |
| *Note*. Interaction effects controlling for age, gender, race/ethnicity, education. Gender was coded as 1 = Woman, 2 = Man, 3 = Non-binary/Other. Race/ethnicity was coded as 1 = White, 2 = Black/African American, 3 = Native American/Eskimo/Aleut, 4 = Hispanic/Latinx, 5 = Asian/Asian-American, 6 = Native Hawaiian or Pacific Islander, 7 = Biracial/Multiracial, 8 = Other. Education was coded as 1 = Less than high school, 2 = High school diploma or equivalent, 3 = Some college, but no degree, 4 = Associate degree, 5 = Bachelor’s degree, 6 = Master’s degree, 7 = Doctorate or professional degree such as JD/MD. Age was entered as a continuous variable. Bold indicates statistical significance (p < .05). SE = standard error; b = unstandardized regression coefficient. Natural log values for comfort eating were used in the analyses. | | | | | | | |
|  |  |  |  |  |  |  |  |
|  |  |  |  |  |  |  |  |
|  |  |  |  |  |  |  |  |
|  |  |  |  |  |  |  |  |

| **Table 4** |  |  |  |  |  |  |  |
| --- | --- | --- | --- | --- | --- | --- | --- |
| *Gender x Weight Stigma Interaction Effect* | |  |  |  |  |  |  |
|  | *b* | *SE* | *t* | 95% CI of *b* | *p* |  |  |
| Sleep disturbance |  |  |  |  |  |  |  |
| Weight Stigma | 0.26 | 0.03 | 7.65 | [0.20, 0.33] | **<.001** |  |  |
| Gender | -0.13 | 0.05 | -2.44 | [-0.24, -0.03] | **.015** |  |  |
| Gender x Weight Stigma | -0.06 | 0.06 | -0.92 | [-0.18, 0.07] | .360 |  |  |
| Age | -0.01 | 0.002 | -5.22 | [-0.01, -0.01] | **<.001** |  |  |
| Race/Ethnicity | -0.0001 | 0.02 | -0.01 | [-0.04, 0.04] | .995 |  |  |
| Education | -0.05 | 0.02 | -3.05 | [-0.09, -0.02] | **.002** |  |  |
| BMI | 0.001 | 0.004 | 0.21 | [-0.01, 0.01] | .832 |  |  |
| Alcohol use |  |  |  |  |  |  |  |
| Weight Stigma | 0.30 | 0.10 | 3.11 | [0.11. 0.49] | **.002** |  |  |
| Gender | 0.90 | 0.15 | 5.93 | [0.60, 1.20] | **<.001** |  |  |
| Gender x Weight Stigma | -0.06 | 0.17 | -0.33 | [-0.40, 0.28] | .743 |  |  |
| Age | -0.03 | 0.01 | -5.57 | [-0.04, -0.02] | **<.001** |  |  |
| Race/Ethnicity | 0.02 | 0.06 | 0.30 | [-0.10, 0.13] | .766 |  |  |
| Education | 0.10 | 0.05 | 1.97 | [0.001, 0.20] | **.049** |  |  |
| BMI | -0.03 | 0.01 | -2.73 | [-0.05, -0.01] | **.007** |  |  |
| Disordered eating |  |  |  |  |  |  |  |
| Weight Stigma | 0.34 | 0.02 | 18.57 | [0.31, 0.38] | **<.001** |  |  |
| Gender | -0.14 | 0.03 | -4.84 | [-0.20, -0.08] | **<.001** |  |  |
| Gender x Weight Stigma | -0.04 | 0.03 | -1.11 | [-0.10, 0.03] | .266 |  |  |
| Age | -0.01 | 0.001 | -6.22 | [-0.01, -0.004] | **<.001** |  |  |
| Race/Ethnicity | -0.01 | 0.01 | -0.69 | [-0.03, 0.01] | .488 |  |  |
| Education | 0.03 | 0.01 | 2.96 | [0.01, 0.05] | **.003** |  |  |
| BMI | 0.01 | 0.002 | 3.61 | [0.003, 0.01] | **<.001** |  |  |
| Comfort eating |  |  |  |  |  |  |  |
| Weight Stigma | 0.32 | 0.04 | 8.82 | [0.25, 0.39] | **<.001** |  |  |
| Gender | 0.02 | 0.06 | 0.29 | [-0.10, 0.13] | .772 |  |  |
| Gender x Weight Stigma | 0.06 | 0.07 | 0.97 | [-0.07, 0.19] | .334 |  |  |
| Age | -0.01 | 0.002 | -4.90 | [-0.01, -0.006] | **<.001** |  |  |
| Race/Ethnicity | -0.004 | 0.02 | -0.17 | [-0.05, 0.04] | .866 |  |  |
| Education | 0.05 | 0.02 | 2.63 | [0.01, 0.09] | **.009** |  |  |
| BMI | 0.01 | 0.004 | 3.50 | [0.01, 0.02] | **.001** |  |  |
| Physical activity |  |  |  |  |  |  |  |
| Weight Stigma | -0.04 | 0.05 | -0.79 | [-0.13, 0.06] | .428 |  |  |
| Gender | 0.18 | 0.08 | 2.45 | [0.04, 0.33] | **.015** |  |  |
| Gender x Weight Stigma | 0.10 | 0.09 | 1.13 | [-0.07, 0.27] | .257 |  |  |
| Age | -0.01 | 0.003 | -2.82 | [-0.01, -0.002] | **.005** |  |  |
| Race/Ethnicity | -0.11 | 0.03 | -3.72 | [-0.16, -0.05] | **<.001** |  |  |
| Education | 0.09 | 0.02 | 3.80 | [0.05, 0.14] | **<.001** |  |  |
| BMI | -0.04 | 0.01 | -6.71 | [-0.05, -0.03] | **<.001** |  |  |
| *Note.* Interaction effects controlling for age, race/ethnicity, education, BMI. Gender was coded as 1 = Woman, 2 = Man, 3 = Non-binary/Other. Race/ethnicity was coded as 1 = White, 2 = Black/African American, 3 = Native American/Eskimo/Aleut, 4 = Hispanic/Latinx, 5 = Asian/Asian-American, 6 = Native Hawaiian or Pacific Islander, 7 = Biracial/Multiracial, 8 = Other. Education was coded as 1 = Less than high school, 2 = High school diploma or equivalent, 3 = Some college, but no degree, 4 = Associate degree, 5 = Bachelor’s degree, 6 = Master’s degree, 7 = Doctorate or professional degree such as JD/MD. Age and BMI were entered as continuous variables. Bold indicates statistical significance (p < .05). SE = standard error; b = unstandardized regression coefficient. Natural log values for comfort eating were used in the analyses. | | | | | | | |

**Online Supplementary Materials 4**

**Validation of composite weight stigma measure**

The present study used a 2-item composite measure of daily anticipated and experienced weight stigma. Anticipated weight stigma was assessed on a 4-point Likert scale (1 = Not at all to 4 = Often): “In your day-to-day life, how often are you concerned about or worried you will be negatively stereotyped or mistreated because of your weight?” Experienced weight stigma was assessed on a 4-point Likert scale (1 = Not at all to 4 = Often): “In your day-to-day life, how often are you treated with less respect, harassed, or discriminated against because of your weight?”

The two weight stigma items were developed based on a previous questionnaire (Hunger & Major, 2015). We conducted a validation study in 217 separate participants to validate the single items and composite weight stigma measure. Pearson correlations were performed to test associations between the single-item anticipated weight stigma and experienced weight stigma measures against two validated measures of weight stigma: the Fear of Enacted subscale of the Weight Self Stigma Questionnaire (*r* = .689, *p* < .001) and the Stigmatizing Situations Inventory-Brief (SSI-B) (*r* = .702, *p <* .001), respectively. The two-item composite measure was also validated against the SSI-B (*r* = .725, *p* < .001) and the Fear of Enacted subscale (*r* = .704, *p* < .001). We used standard criteria to evaluate the magnitude of the obtained correlations (i.e., 0.1 = small, 0.3 = moderate, 0.5 = large). Our analyses revealed that the individual weight stigma items (i.e., anticipated and experienced), as well as the composite 2-item weight stigma score, showed large correlations with validated weight stigma questionnaires.

Additional psychometric assessments will be needed to assess test-retest reliability. Our preliminary results indicate that the 2-item composite weight stigma measure is a valid questionnaire to assess weight stigma when survey constraints are a concern.
